# Economic Evaluation of Extended-Release Buprenorphine for Persons With Opioid Use Disorder

**DOI:** 10.1001/jamanetworkopen.2023.29583

**Published:** 2023-09-13

**Authors:** Juliet M. Flam-Ross, Elizabeth Marsh, Michelle Weitz, Alexandra Savinkina, Bruce R. Schackman, Jianing Wang, R. W. M. A. Madushani, Jake R. Morgan, Joshua A. Barocas, Alexander Y. Walley, Stavroula A. Chrysanthopoulou, Benjamin P. Linas, Sabrina A. Assoumou

**Affiliations:** 1 Section of Infectious Diseases, Boston Medical Center, Boston, Massachusetts; 2 Now with London School of Hygiene and Tropical Medicine, London, United Kingdom; 3 Section of Infectious Diseases, Boston University Chobanian & Avedisian School of Medicine, Boston, Massachusetts; 4 Yale School of Public Health, New Haven, Connecticut; 5 Department of Population Health Sciences, Weill Cornell Medical College, New York, New York; 6 Section of General Internal Medicine and Infectious Diseases, University of Colorado Anschutz Medical Campus, Aurora; 7 Department of Medicine, Section of General Internal Medicine, Boston Medical Center, Boston, Massachusetts; 8 Department of Biostatistics, Brown University School of Public Health, Brown University, Providence, Rhode Island; 9 Boston University School of Public Health, Boston, Massachusetts

## Abstract

**Importance:**

In 2017, the US Food and Drug Administration (FDA) approved a monthly injectable form of buprenorphine, extended-release buprenorphine; published data show that extended-release buprenorphine is effective compared with no treatment, but its current cost is higher and current retention is lower than that of transmucosal buprenorphine. Preliminary research suggests that extended-release buprenorphine may be an important addition to treatment options, but the cost-effectiveness of extended-release buprenorphine compared with transmucosal buprenorphine remains unclear.

**Objective:**

To evaluate the cost-effectiveness of extended-release buprenorphine compared with transmucosal buprenorphine.

**Design, Setting, and Participants:**

This economic evaluation used a state transition model starting in 2019 to simulate the lifetime of a closed cohort of individuals with OUD presenting for evaluation for opioid agonist treatment with buprenorphine. The data sources used to estimate model parameters included cohort studies, clinical trials, and administrative data. The model relied on pharmaceutical costs from the Federal Supply Schedule and health care utilization costs from published studies. Data were analyzed from September 2021 to January 2023.

**Interventions:**

No treatment, treatment with transmucosal buprenorphine, or treatment with extended-release buprenorphine.

**Main Outcomes and Measures:**

Mean lifetime costs per person, discounted quality-adjusted life-years (QALYs), and incremental cost-effectiveness ratios (ICERs).

**Results:**

The simulated cohort included 100 000 patients with OUD receiving (61% male; mean [SD] age, 38 [11] years) or not receiving medication treatment (58% male, mean [SD] age, 48 [18] years). Compared with no medication treatment, treatment with transmucosal buprenorphine yielded an ICER of $19 740 per QALY. Compared with treatment with transmucosal buprenorphine, treatment with extended-release buprenorphine yielded lower effectiveness by 0.03 QALYs per person at higher cost, suggesting that treatment with extended-release buprenorphine was dominated and not preferred. In probabilistic sensitivity analyses, treatment with transmucosal buprenorphine was the preferred strategy 60% of the time. Treatment with extended-release buprenorphine was cost-effective compared with treatment with transmucosal buprenorphine at a $100 000 per QALY willingness-to-pay threshold only after substantial changes in key parameters.

**Conclusions and Relevance:**

In this economic evaluation of extended-release buprenorphine compared with transmucosal buprenorphine for the treatment of OUD, extended-release buprenorphine was not associated with efficient allocation of limited resources when transmucosal buprenorphine was available. Future initiatives should aim to improve retention rates or decrease costs associated with extended-release buprenorphine.

## Key Points

**Question:**

When considering extended-release buprenorphine or transmucosal buprenorphine for treatment for opioid use disorder, which therapy provides the best value for money?

**Findings:**

This economic evaluation, which used a state transition model to simulate a population with opioid use disorder and included cohorts of 100 000 simulated individuals in each category of intervention, found that when compared with no medication treatment, treatment with transmucosal buprenorphine yielded an incremental cost-effectiveness ratio of $19 740 quality-adjusted life-years gained. In comparison, treatment with extended-release buprenorphine yielded lower effectiveness by 0.03 quality-adjusted life-years per person at higher cost, suggesting that treatment with extended-release buprenorphine was dominated and therefore not preferred.

**Meaning:**

These results suggest that at current medication cost and retention rates, extended-release buprenorphine was not associated with efficient allocation of limited resources when transmucosal buprenorphine was available; future initiatives should aim to improve retention rates and decrease cost.

## Teaser

This economic evaluation examines the cost-effectiveness of extended-release buprenorphine compared with transmucosal buprenorphine.

## Introduction

The US recorded 83 000 opioid-related overdose deaths in 2022.^1^ Evidence shows that medications for opioid use disorder (MOUD) are a tool that helps reduce opioid-related overdose mortality.^2,3,4^ Although MOUD are highly effective, many eligible individuals are not receiving them due in part to the limited number of prescribers, financial accessibility, restrictions on methadone care, and stigma.^5,6^ Although access to and uptake of MOUD has risen, the increase has not been substantial enough to reverse the trajectory of opioid overdose–related death rates.^7,8^

MOUD currently approved by the Food and Drug Administration (FDA) are buprenorphine, methadone, and naltrexone. Buprenorphine is available as transmucosal buprenorphine, which is most commonly taken daily as a sublingual film and extended-release buprenorphine, which is administered as a monthly injection.^9,10^ According to Federal Supply Schedule data from 2022, monthly costs are $196 for transmucosal buprenorphine and $1136 for extended-release buprenorphine.^11^ In addition, a study of commercially insured individuals focusing on persons who had initiated extended-release buprenorphine within 13 months after FDA approval found lower retention on treatment for extended-release buprenorphine compared with other forms of MOUD.^12,13^ Nevertheless, qualitative research suggests some notable benefits associated with extended-release buprenorphine, including a decrease in stigma, ability to lead a “normal life,” and increased convenience.^14,15^ These factors all suggest a potential for improved retention on extended-release buprenorphine for later adopters.^14,15^

Given the urgency of opioid overdose–related deaths, it would be useful for patients, clinicians, and policy makers to know the relative cost-effectiveness of extended-release buprenorphine vs transmucosal buprenorphine. Recent changes to address barriers to prescribing buprenorphine also make the current analysis timely.^16^ Therefore, we used a computer simulation model to investigate the cost-effectiveness of extended-release buprenorphine when compared with transmucosal buprenorphine.^17^ We also explored under which conditions extended-release buprenorphine would not provide a good value for money given its known limitations.

## Methods

This economic evaluation was deemed exempt from review and informed consent by the institutional review board at the Boston University Medical Campus because the simulation modeling used either previously published data from the literature or publicly available data. We followed the Consolidated Health Economic Evaluation Reporting Standards (CHEERS) reporting guideline.

### Model Overview and Strategies

We conducted an economic evaluation using the Researching Effective Strategies to Prevent Opioid Death (RESPOND) model to evaluate the cost-effectiveness of extended-release buprenorphine. RESPOND is a cohort-based, state transition model of OUD natural history and treatment in Massachusetts (eFigure 1 in Supplement 1).^18^ Additional details on the model are included in previous work^18,19^ and in the eAppendix, eFigure 2, eTable 1, and eTable 2 in Supplement 1. We simulated the lifetime of a closed cohort of 100 000 individuals with OUD in Massachusetts starting in 2019. We modeled a base case of 3 strategies: (1) no medication treatment: individuals do not access MOUD or medically managed withdrawal (detoxification); (2) treatment with transmucosal buprenorphine: individuals start receiving transmucosal buprenorphine; if they are not retained, they transition between no treatment, extended-release buprenorphine, methadone, naltrexone, and detoxification; (3) treatment with extended-release buprenorphine: individuals start receiving extended-release buprenorphine; if they are not retained, they transition between the same aforementioned options.

While our goal was to study buprenorphine formulations, individuals in the simulation model transition between receiving and not receiving buprenorphine to reflect that in the real world they can access other treatment. Although data show that detoxification fails to reduce risk of relapse and overdose, we included it to reflect its real-world use.^20^ As we will describe in more detail later, we do not assume that patients on MOUD are abstinent from drugs.

### Model Structure

We utilized RESPOND to simulate the base case over a lifetime. Weekly transitions were used.^21,22^

#### Natural History of OUD

The model simulated transitions between 4 health states: active injection opioid use; active noninjection opioid use; nonactive injection opioid use; and nonactive noninjection opioid use. We defined active use as use in the past week. There was bidirectional movement between active and nonactive states. In active use, the rate of overdose was higher among people who inject opioids.

#### Care Delivery

Each week, a subset of individuals accessed treatment and detoxification. Individuals receiving MOUD experience bidirectional transitions between active and nonactive states, representing the reality that some patients who are taking MOUD use opioids, but the net balance of movement favors nonactive use.^23^

These individuals also faced a risk of ceasing MOUD. This population entered a 4-week posttreatment state, during which the rates of return to active use and overdose were higher than those for individuals not receiving treatment. In contrast, receiving MOUD also had an independent effect of lowering the rate of overdose among those who were actively using drugs in the model.

#### Population Dynamics

We ran a closed cohort without adding or removing members from the simulation (except for death) to replicate the experience of an observational cohort study. The total number of individuals in the cohort decreased until everyone had died. Initial cohorts were stratified by age, sex, and OUD status. These data were informed by calibrated proportions of individuals in each category from 2012.^24^

#### Mortality

Among individuals in active use states, there was a risk of overdose in every cycle. Overdose incidence was considered as a function of both age and mode of drug consumption (injection or noninjection). A proportion of these overdoses were considered as overdose deaths. We also used a competing risks approach to adjust for death from other causes using standardized mortality ratios (SMRs).

#### Costs and Utilities

We used MOUD pharmaceutical costs, health care utilization costs, and treatment utilization costs in the model. We stratified the latter 2 (health care utilization costs and treatment utilization costs) by demographic variables. Health care utilization costs accounted for costs associated with prescribing the medication, such as clinical visits. Treatment utilization costs described costs other than the cost of the treatment itself. Costs were reported from the health care system perspective. The model also included demographic-stratified utilities.

### Model Data

The epidemiology of OUD and OUD treatment seeking, including the prevalence of drug use, the incidence of overdose, the rate of treatment seeking, and retention in OUD care were specific to the jurisdiction being simulated. The primary data source for these parameters was the Massachusetts Public Health Data Repository (PHD), which is a longitudinal records database that links individual level data across more than 29 sources.^24,25^ In contrast, we assumed that parameters that simulated the natural history of OUD and the efficacy of pharmaceuticals were generalizable and used data from clinical trials and medical literature. In some cases, data for extended-release buprenorphine were not available and we used estimates based on transmucosal buprenorphine or naltrexone. We summarized our inputs in Table 1.^4,11,24,26,27,28,29,30,31,32,33,34,35,36^

**Table 1.** Model Inputs

Parameter	Baseline value^a^	Range evaluated	Source
Population demographics and epidemiology			
Male, proportion of total people at baseline	Strategy 1: 0.58	Strategy 1: 0.47-0.70	Massachusetts Department of Public Health,^26^ 2017
	Strategies 2 and 3: 0.61	Strategies 2 and 3: 0.49-0.73	
Mean age, y	Strategy 1: 48.21	Strategy 1: 38.66-58.16	Massachusetts Department of Public Health,^26^ 2017
	Strategies 2 and 3: 38.42	Strategies 2 and 3: 28.45-48.42	
Injection drug use, proportion of total people at baseline	0.25	0.20-0.30	SAMHSA,^27^ 2013
Active drug use, proportion of total people at baseline	Strategy 1: 0.91	Strategy 1: 0.73-1.00	Cedarbaum et al,^28^ 2018; Murphy et al,^29^ 2018
	Strategy 2: 0.25	Strategy 2: 0.20-0.28	
	Strategy 3: 0.25	Strategy 3: 0.20-0.30	
SMR for injection drug use	5.07	4.06-6.09	Massachusetts Department of Public Health,^26^ 2017; US Census Bureau,^30^ 2013
SMR for noninjection drug use	2.05	1.64-2.46	Massachusetts Department of Public Health,^26^ 2017; US Census Bureau,^30^ 2013
Transition to MOUD treatment and detoxification, monthly rate per 1000 people			
Transmucosal buprenorphineand extended-release buprenorphine	8.41	6.81-10.00	Massachusetts Department of Public Health,^26^ 2017
Methadone	2.40	1.92-2.92	Massachusetts Department of Public Health,^26^ 2017
Naltrexone (injectable)	1.08	0.88-1.32	Massachusetts Department of Public Health,^26^ 2017
Detoxification	6.80	5.20-8.00	Massachusetts Department of Public Health,^24^2020
Retained on MOUD treatment, proportion at 6 mo			
Transmucosal buprenorphine	0.47	0.45-0.47	IBM,^31^ 2021
Extended-release buprenorphine	0.29	0.24-0.33	IBM,^31^ 2021
Methadone	0.66	0.64-0.69	IBM,^31^ 2021
Naltrexone (injectable)	0.32	0.30-0.34	IBM,^31^ 2021
Overdose, monthly rate per 1000 people			
No treatment	6.76	5.40-8.00	Massachusetts Department of Public Health,^26^ 2017
Transmucosal buprenorphineand extended-release buprenorphine	2.72	2.20-3.28	Morgan et al,^32^ 2019
Methadone	5.08	4.04-6.08	Sordo et al,^4^ 2017
Naltrexone (injectable)	5.80	4.68-7.00	Morgan et al,^32^ 2019
Fatal overdoses, proportion of total overdoses			
All treatment states	0.14	0.11-0.16	Massachusetts Department of Public Health,^26^ 2017
Pharmaceutical cost			
Transmucosal buprenorphine (16 mg, daily), $	49	39-58	US Department of Veterans Affairs,^11^ 2021
Extended-release buprenorphine (100 mg or 300 mg, injection, monthly), $	284	227-341	US Department of Veterans Affairs,^11^ 2021
Methadone (80 mg, daily), $	4	3-5	US Department of Veterans Affairs,^11^ 2021
Naltrexone (380 mg, injection, monthly), $	303	242-363	US Department of Veterans Affairs,^11^ 2021
Treatment utilization cost^b^			
Transmucosal buprenorphine, $	65	52-72	Expert opinion^c^
Extended-release buprenorphine and naltrexone (injectable), $	24	19-29	Expert opinion^c^
Methadone, $	123	99-148	NIDA,^33^ 2021
Detoxification, $	2863	2290-3436	McCollister et al,^34^2018; Murphy et al,^29^, 2019
Overdose cost, weekly			
Fatal overdose cost, $	4557	3646-5469	Coffin et al,^35^ 2017; Jiang et al,^36^ 2017
Nonfatal overdose cost, $	858	686-1030	Coffin et al,^35^ 2017; Jiang et al,^36^ 2017

Abbreviations: MOUD, medications for opioid use disorder; NIDA, the National Institute of Drug Abuse; SAMHSA, Substance Abuse and Mental Health Services Administration; SMR, standardized mortality ratio.

^a^ Values for all strategies are the same unless otherwise noted. Where noted, strategy 1 is no medication treatment; strategy 2 is treatment with transmucosal buprenorphine; strategy 3 is treatment with extended-release buprenorphine.

^b^ Treatment costs include any costs required in addition to the pharmaceutical cost for administration of the drug.

^c^ For parameters that were not available from the literature, addiction medicine specialists listed as coauthors for the current study were consulted for reasonable estimates. Extensive sensitivity analyses were then used to assess the robustness of all parameters in the model.

#### Demography and Opioid Use

We estimated the demographics of the OUD population in Massachusetts using results from a capture-recapture analysis that utilized data from the Massachusetts PHD.^25^ We also used data from multiple cross-sectional surveys to estimate the proportion of people in different health states for each cohort.^27,28,37^

The mean age (SD) in the no medication treatment strategy was 48 (18) years, and in the treatment strategies it was 38 (11) years.^25^ The proportion of male individuals at baseline was 58% in the no medication treatment strategy, and 61% in the treatment strategies.^25^

#### Overdose

The monthly rate of overdose among people not receiving treatment is 6.76 per 1000 people.^24,26^ The monthly rate of overdose among individuals treated with transmucosal buprenorphine or extended-release buprenorphine is 2.72 per 1000 people.^4,32^ Among those who experience an overdose, 14% die, which is the same proportion for both injection and noninjection use.^24,26^ We used data from the Massachusetts PHD to determine overdose rates for individuals not on treatment and proportion of fatal overdoses.^24,26^ We modeled overdose rates while receiving treatment using data from cohort studies.^4,32^

#### OUD Treatment Modalities

We used weekly probabilities of MOUD treatment retention with data from the IBM Watson MarketScan Commercial Claims Database, a nationally representative data set of US commercially insured individuals.^31^ Retention on transmucosal buprenorphine is 47% at 6 months, whereas retention on extended-release buprenorphine is 29% at 6 months.^31^

#### Costs

We used pharmaceutical costs from the Federal Supply Schedule and estimated health care utilization costs and treatment utilization costs from published studies.^11,29,33,34^ The weekly pharmaceutical cost of extended-release buprenorphine is $284, while the cost of transmucosal buprenorphine is $49. We included the overdose costs from published studies in the model.^35,36^

#### Utilities

We assigned utility weights, representing general well-being, based on age, sex, treatment state, and OUD status (eTable 1 in Supplement 1). We used utility weights from published studies.^29,38^

### Statistical Analysis

First, we used the model to simulate the base case clinical progression. Outcomes from the simulation included life expectancy, life-years, quality-adjusted life-years (QALYs) using utility weights, and lifetime costs from the health sector perspective. We reported QALYs as their increase from the base level age. We discounted costs and QALYs by 3% annually and used a minimal approach for estimating utilities, meaning that when multiple conditions affecting utility are experienced simultaneously, we assigned the utility weight associated with the worst condition.^39^ When necessary, we adjusted costs for inflation to 2019 USD. We compared all strategies using incremental cost-effectiveness ratios (ICERs) using a willingness-to-pay threshold of $100 000 per QALY, which we calculated as the incremental cost divided by the incremental change in QALYs of the next most costly strategy.^39^ We considered strategies to be dominated if they were more costly and less effective than the next most costly strategy.

We conducted deterministic sensitivity analyses on all inputs by varying point estimates over a feasible range to evaluate the effects of uncertainty around model parameters (Table 1). We also conducted sensitivity analyses on costs and retention to identify threshold values for cost-effectiveness. Additionally, we performed a sensitivity analysis on utility weights using the multiplicative method, where the utility weights are multiplied.^39^

We conducted probabilistic sensitivity analyses by defining probability density functions (distributions) for overdose rates, treatment initiation, fatal overdose rate, SMRs, transitions between treatments (including retention and uptake), costs, and utility. We obtained the distribution of values (including shape and mean) from published literature sources and existing data. We repeated each simulation 10 000 times, using a different random value from the feasible range for every input. Statistical analysis was performed using R Studio version 3.5.1 (R Project for Statistical Computing) from September 2021 to January 2023.

## Results

### Base Case

A cohort of 100 000 simulated individuals was used in each category of intervention (among no medication treatment cohort: 58% were male and mean [SD] age was 48 [18] years; among treatment cohort: 61% were male and mean [SD] age was 38 [11] years) (Table 1). When compared with no medication treatment, treatment with transmucosal buprenorphine was associated with an increased life expectancy (27.44 vs 20.54 life-years) (Table 2). This increase represents an additional 3.22 discounted QALYs, and raised discounted lifetime costs per person ($304 700 vs $241 070). When compared with no medication treatment, treatment with transmucosal buprenorphine had an ICER of $19 740 per QALY, which is below the commonly cited willingness-to-pay threshold of $100 000 per QALY.

**Table 2.** Results of Base Case Analysis

Strategy	Annual fatal overdose rate per 1000 people^a^	Remaining undiscounted LYs per person^b^	Total discounted cost per person, $^b^	Change in discounted cost per person, $^b^	Remaining discounted QALYs per person^b^	Change in remaining discounted QALYs per person^b^	ICER ($/LY)^b^	ICER ($/QALY)^b^
No medication treatment strategy	7.77	20.54	241 070	NA	8.32	NA	NA	NA
Treatment with transmucosal buprenorphine strategy	6.27	27.44	304 700	63 630	11.55	3.22	18 380	19 740
Treatment with extended-release buprenorphine strategy	6.42	27.39	308 700	4000	11.52	−0.03	Dominated^c^	Dominated^c^

Abbreviations: ICER, incremental cost-effectiveness ratio; LY, life-years; NA, not applicable; QALY, quality-adjusted life-years.

^a^ Calculated over the first 10 years of the simulation.

^b^ Costs and ICERs were rounded to nearest $10 and QALYs and LYs to nearest 0.01.

^c^ A strategy was considered dominated when costing more and achieving a lower QALY than the next least expensive strategy.

When treatment with extended-release buprenorphine was compared with treatment with transmucosal buprenorphine, it had fewer remaining undiscounted life-years per person (27.44 vs 27.39 life-years), a decrease of 0.03 QALYs, and an increase in discounted lifetime costs per person ($308 700 vs $304 700). The treatment with extended-release buprenorphine strategy was therefore dominated.

### Sensitivity Analyses

#### Deterministic Sensitivity Analyses

##### Pharmaceutical Cost

The cost-effectiveness of treatment with extended-release buprenorphine varied depending on changes in extended-release buprenorphine pharmaceutical cost (Table 3). After applying an 80% decrease in pharmaceutical cost, corresponding to a monthly pharmaceutical cost of $57, the following observations were noted. The total cost for treatment with extended-release buprenorphine was lower than that for treatment with transmucosal buprenorphine ($293 040 vs $293 730). When compared with no medication treatment, treatment with extended-release buprenorphine was associated with an ICER of $16 300 per QALY gained. In addition, when compared with treatment with extended-release buprenorphine, treatment with transmucosal buprenorphine had an ICER of $19 760 per QALY gained.

**Table 3.** Results of Deterministic Sensitivity Analyses

Strategy	Annual overdose rate per 1000 people^a^	Remaining undiscounted LYs per person^b^	Total discounted cost per person, $^b^	Change in discounted cost per person, $^b^	Remaining discounted QALYs per person^b^	Change in remaining discounted QALYs per person^b^	ICER ($/LY)^b^	ICER ($/QALY)^b^
**Sensitivity analysis: 80% decreased pharmaceutical cost**								
No medication treatment strategy	7.77	20.54	241 070	NA	8.33	NA	NA	NA
Treatment with extended-release buprenorphine strategy	6.53	27.39	293 040	51 970	11.52	3.19	15 140	16 300
Treatment with transmucosal buprenorphine strategy	6.49	27.44	293 730	680	11.55	0.03	23 410	19 760
**Sensitivity analysis: 191% increased retention**								
No medication treatment strategy	7.77	20.54	241 070	NA	8.33	NA	NA	NA
Treatment with transmucosal buprenorphine strategy	5.10	28.26	338 480	97 410	12.07	3.75	25 560	25 990
Treatment with extended-release buprenorphine strategy	4.27	28.57	358 280	19 800	12.28	0.20	118 610	97 840

Abbreviations: ICER, incremental cost-effectiveness ratio; LY, life-years; QALY, quality-adjusted life-years.

^a^ Calculated over the first 10 years of the simulation.

^b^ Costs and ICERs were rounded to nearest $10 and QALYs and LYs to nearest 0.01.

##### Retention

**Figure 1.**
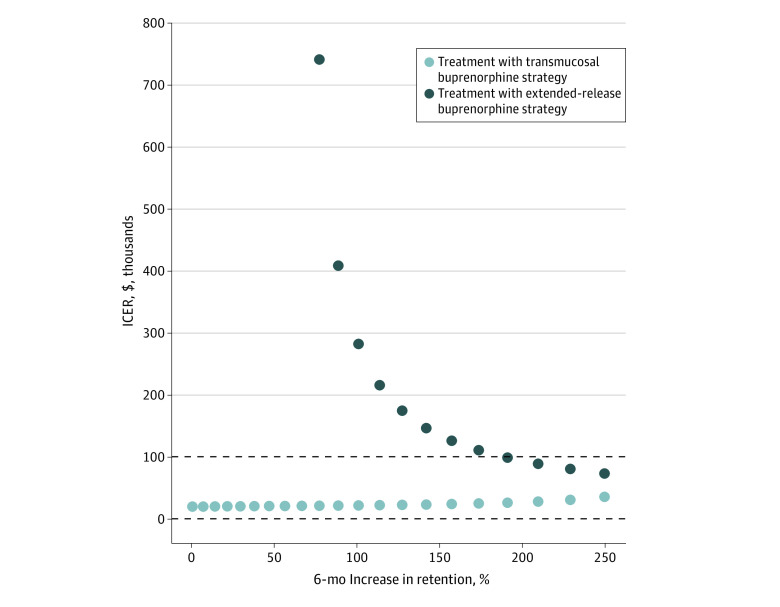
One-Way Sensitivity Analysis of Retention on Medications for Opioid Use Disorder The figure shows the cost-effectiveness of treatment with transmucosal buprenorphine and treatment with extended-release buprenorphine strategies as the 6-month rate of retention is increased. The dotted line at the incremental cost-effectiveness ratio (ICER) of $100 000 represents a threshold of $100 000 per quality-adjusted life years (QALY) willingness-to-pay and the dotted line at $0 represents the lowest value at which any strategy is not dominated. Any dots shown between the 2 sets of dotted lines are considered to be cost-effective strategies.

The cost-effectiveness of treatment with extended-release buprenorphine was also sensitive to changes in retention on extended-release buprenorphine (Table 3; Figure 1). When we increased the 6-month retention rate from 29% to 83%, or an overall increase of 191%, QALYs associated with treatment with extended-release buprenorphine were slightly higher than that of treatment with transmucosal buprenorphine (12.28 QALYs vs 12.07 QALYs) and the ICER was $25 990 per QALY gained for treatment with transmucosal buprenorphine when compared with no medication treatment (eTable 1 in Supplement 1). As for the treatment with extended-release buprenorphine strategy, comparison with treatment with transmucosal buprenorphine had an ICER of $97 840 per QALY gained.

##### Two-Way Cost and Retention

**Figure 2.**
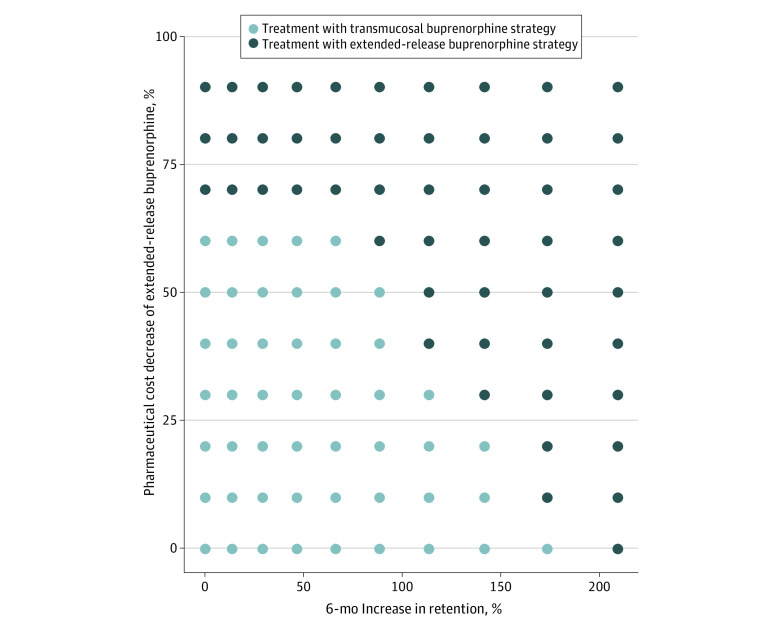
Two-Way Deterministic Sensitivity Analysis of Pharmaceutical Cost and Retention for Extended-Release Buprenorphine Cost-Effectiveness Plot This figure represents the cost-effective strategy at a $100 000 per quality-adjusted life years (QALY) willingness-to-pay threshold when we vary both retention and cost for extended-release buprenorphine at the same time in increments of 5%. The values in the upper right side of the figure, where the dots turn to darkest, are combinations of retention increases and cost reductions resulting in the treatment with extended-release buprenorphine strategy no longer being dominated.

The cost-effectiveness of treatment with extended-release buprenorphine changed in response to simultaneous changes in the pharmaceutical cost and extended-release buprenorphine retention (2-way sensitivity analysis). In Figure 2, we show extended-release buprenorphine retention values and pharmaceutical costs for which treatment with extended-release buprenorphine was cost-effective when compared with treatment with transmucosal buprenorphine. For example, a combination of a 40% decrease in cost and an approximately 2 times improvement in retention led to extended-release buprenorphine as a good value for money. Given that the current study is modeling a cohort of individuals in 2019, Figure 2 may be particularly helpful to understand the cost-effectiveness of interventions considered as cost and retention continue to fluctuate.

#### Other Sensitivity Analyses

Findings were robust to variations in demographic characteristics, epidemiological model inputs, costs, and utilities. Results for key ranges evaluated are included in Table 1. We also included sensitivity analysis results on the type of utility used (multiplicative vs minimal approach) in eTable 2 in Supplement 1. Remaining ranges are included in the eAppendix in Supplement 1.

#### Probabilistic Sensitivity Analysis

We used probabilistic sensitivity analyses to incorporate uncertainty. Assuming a willingness to pay of $100 000 per QALY gained, treatment with transmucosal buprenorphine was the optimal strategy in 60% of simulations, whereas treatment with extended-release buprenorphine was cost-effective in the remaining 40%.

## Discussion

This economic evaluation found that when using currently available cost and retention data, extended-release buprenorphine was not a cost-effective strategy when transmucosal buprenorphine was available. This finding was robust over a reasonable range of variations in key parameters, and we also found that substantial changes in pharmaceutical cost and retention rates would be necessary for extended-release buprenorphine to become a good value for money.

Our findings do not imply that extended-release buprenorphine has no role in the market. Previous studies show that extended-release buprenorphine has the potential to be a helpful addition to existing forms of MOUD. Injectables include a consistent dose of medication and reduced burden of daily medication. Extended-release buprenorphine may decrease stigma that is associated with daily dosing of medication.^14^ In addition, extended-release buprenorphine may be particularly useful for populations at high risk for overdose fatalities, including survivors of opioid overdose.^40^ Furthermore, a study showed that almost a quarter of respondents preferred extended-release buprenorphine when given a choice between transmucosal buprenorphine, extended-release buprenorphine, or a buprenorphine implant.^41^ Although we found that at current cost and retention rates, extended-release buprenorphine was not preferred when compared with transmucosal buprenorphine, not all patients are willing to initiate transmucosal buprenorphine. Our study specifically contributes to the literature by estimating changes in key parameters such as cost and retention that would lead to extended-release buprenorphine becoming a cost-effective option when other buprenorphine formulations are available. In addition, the current analysis is especially timely given that another buprenorphine extended-release subcutaneous injection drug was recently approved by the FDA.^42^ The availability of new drugs might lead to more competition and a decreased price.^43^ This situation might be akin to what was observed with another health condition, hepatitis C. In this particular case, our team and others found that some drugs that were not a good value for money at higher prices, later became acceptable options when prices were lowered.^44,45^ As opioid overdoses continue to surge nationwide, unabated and likely worsened by the COVID-19 pandemic, it is crucial to develop a broad menu of effective MOUD for a population with diverse needs.

Although our study found that extended-release buprenorphine may not currently be a cost-effective option when transmucosal buprenorphine is available, we also found that if this medication’s cost were decreased by 80%, or retention over 6 months were to increase from 29% to 83%, this conclusion may change. Given that implementing these changes might require substantial adjustments, our 2-way sensitivity analysis shows combinations of cost and retention values for which extended-release buprenorphine may become a cost-effective option when transmucosal buprenorphine is available.

We found that our results were robust with variations over a wide range of deterministic sensitivity analyses; however, by assigning a distribution around key input parameters in probabilistic sensitivity analyses, we found that extended-release buprenorphine was cost-effective in 40% of the simulations. This result suggests that our findings might be substantially influenced by uncertainty around some baseline parameters such as retention in care. Therefore, additional information on some parameters would improve estimates and might modify our conclusions. Addressing the overdose crisis will require a variety of options in the OUD treatment toolbox and extended-release buprenorphine has the potential to improve outcomes.

### Limitations

Our study has limitations. First, available data on extended-release buprenorphine are for patients who were prescribed extended-release buprenorphine within 13 months of its approval. Some parameter estimates, particularly related to retention, may change as later adopters constitute a larger proportion of prescribers. Also, our results may not be applicable outside of the US even though our 2-way analysis of cost and retention may help a diverse range of stakeholders with decisions related to the use of extended-release buprenorphine. We used Massachusetts data because this state has some of the best data on OUD and its complications.^46^ Our results are, therefore, likely applicable to other places in the country with a similar mix of urban and rural communities. Furthermore, our data on utilities for MOUD may not reflect patients’ preferences for one medication over another. Additionally, our use of QALYs may not fully reflect quality of life as experienced by people who use drugs. Nonetheless, they provide a basis for comparing our results with those reported by others in the literature, and we also report life-year results. Next, given that data on nonfatal overdoses are incomplete, the proportion of fatal overdoses might need to be refined. Nevertheless, our estimated overdoses from the calibrated simulation match the overdoses reported in Massachusetts in 2012 from the PHD data set. Also, there are no clear data of counseling interventions effective in combination with buprenorphine to inform detailed simulation of the bidirectional effect of counseling and MOUD.^47^ Additionally, our retention values are from a commercial claims database and therefore they may not be representative of overall retention rates on MOUD.

## Conclusions

In this economic evaluation of extended-release buprenorphine compared with transmucosal buprenorphine for patients with OUD, we found that extended-release buprenorphine was not a cost-effective treatment option when transmucosal buprenorphine was available, and we identified thresholds in cost and MOUD retention that would change the main conclusion of our analysis. The ongoing nationwide surge in opioid overdose deaths underscores the need for a broad menu of effective treatments to address the diverse needs of all patients. Our findings on important thresholds in 2 key parameters, namely cost and retention, might be helpful to policy makers. Future research should evaluate the experience of later extended-release buprenorphine adopters as well as the role of interventions aimed to enhance retention or decrease extended-release buprenorphine cost.

## Notes

Supplement 1. **eAppendix.** Supplemental Materials **eTable 1.** Utility Values (For Active Treatment) **eTable 2.** Multiplicative Utility Sensitivity Analysis on Base Case **eFigure 1.** Model Structure of Researching Effective Strategies to Prevent Opioid Death (RESPOND) **eFigure 2.** Sensitivity of Cost and Quality-Adjusted Life Years for the Extended-Release Buprenorphine Strategy
eReferences

Supplement 2.
Data Sharing Statement
