# Supplementary material for: Economic Evaluation of Extended-Release Buprenorphine for Persons With Opioid Use Disorder
Source: JAMA Netw Open. 2023 Sep 13;6(9):e2329583. doi: 10.1001/jamanetworkopen.2023.29583 (PMC10500382; doi:10.1001/jamanetworkopen.2023.29583)
Supplement: Supplement 2. — Data Sharing Statement [file jamanetwopen-e2329583-s002.pdf]

Data Sharing Statement

Flam-Ross. Economic Evaluation of Extended-Release Buprenorphine for Persons With Opioid Use Disorder. *JAMA Netw Open*. Published September 07, 2023. doi:10.1001/jamanetworkopen.2023.29583

Data
Data available: No

Additional Information
Explanation for why data not available: The data that we employ to build the simulation do not belong to us – we employ secondary data analysis to build the simulation. We therefore cannot share those data. Supplemental materials for the RESPOND model are available at https://www.syndemicslab.org/.
